# TRIM56-mediated production of type I interferon inhibits intracellular replication of *Rickettsia rickettsii*

**DOI:** 10.1128/spectrum.03695-23

**Published:** 2024-02-15

**Authors:** Ruxi Cheng, Chunyu Zhou, Mingliang Zhao, Shan Zhang, Weiqiang Wan, Yonghui Yu, Bohai Wen, Jun Jiao, Xiaolu Xiong, Qin Xu, Xuan OuYang

**Affiliations:** 1 Artemisinin Research Center, Guangzhou University of Chinese Medicine, Guangzhou, Guangdong, China; 2 State Key Laboratory of Pathogen and Biosecurity, Beijing Institute of Microbiology and Epidemiology, Beijing, China; Griffith University–Gold Coast Campus, Gold Coast, Australia

**Keywords:** *Rickettsia rickettsii*, TRIM56, cGAS-STING, ubiquitination, IFN-β, innate immune response

## Abstract

*Rickettsia rickettsii* (*R. rickettsii*), the causative agent of Rocky Mountain spotted fever (RMSF), is the most pathogenic member among *Rickettsia* spp. Previous studies have shown that tripartite motif-containing 56 (TRIM56) E3 ligase-induced ubiquitination of STING is important for cytosolic DNA sensing and type I interferon production to induce anti-DNA viral immunity, but whether it affects intracellular replication of *R. rickettsii* remains uncharacterized. Here, we investigated the effect of TRIM56 on HeLa and THP-1 cells infected with *R. rickettsii*. We found that the expression of TRIM56 was upregulated in the *R. rickettsii*-infected cells, and the overexpression of TRIM56 inhibited the intracellular replication of *R. rickettsii*, while *R. rickettsii* replication was enhanced in the TRIM56-silenced host cells with the reduced phosphorylation of IRF3 and STING and the increased production of interferon-β. In addition, the mutation of the TRIM56 E3 ligase catalytic site impairs the inhibitory function against *R. rickettsii* in HeLa cells. Altogether, our study discovers that TRIM56 is a host restriction factor of *R. rickettsii* by regulating the cGAS-STING-mediated signaling pathway. This study gives new evidence for the role of TRIM56 in the innate immune response against intracellular bacterial infection and provides new therapeutic targets for RMSF.

**IMPORTANCE:**

Given that *Rickettsia rickettsii* (*R. rickettsii*) is the most pathogenic member within the *Rickettsia* genus and serves as the causative agent of Rocky Mountain spotted fever, there is a growing need to explore host targets. In this study, we examined the impact of host TRIM56 on *R. rickettsii* infection in HeLa and THP-1 cells. We observed a significant upregulation of TRIM56 expression in *R. rickettsii*-infected cells. Remarkably, the overexpression of TRIM56 inhibited the intracellular replication of *R. rickettsii*, while silencing TRIM56 enhanced bacterial replication accompanied by reduced phosphorylation of IRF3 and STING, along with increased interferon-β production. Notably, the mutation of the TRIM56’s E3 ligase catalytic site did not impede *R. rickettsii* replication in HeLa cells. Collectively, our findings provide novel insights into the role of TRIM56 as a host restriction factor against *R. rickettsii* through the modulation of the cGAS-STING signaling pathway.

## INTRODUCTION

Spotted fever group (SFG) *Rickettsia* is a group of Gram-negative, obligate intracellular bacteria mainly transmitted by ticks (1), among which *Rickettsia rickettsii* is considered highly pathogenic to humans because it may cause severe disease, Rocky Mountain spotted fever (RMSF) in people. RMSF is characterized by high fever, neurological symptoms, and organ failure (2, 3). If untreated, it can quickly progress into a life-threatening illness in people, with high fatality rates ranging from 30% to 80%. Currently, the mechanism of host innate immune response against *R. rickettsii* infection is largely unknown, which brings barriers to the development of more effective prevention and control measures against RMSF.

Innate immunity is the first line of defense to fight against various pathogen infections. Type I interferons (IFN-I) constitute a critical component of innate immunity and have a nearly universal anti-virus role (4) but have little restricted effect on facultative bacterial pathogens (5). The effect of IFN-I on obligate cytosolic bacterial pathogens, including *Rickettsia* species, has been reported in a few studies. Studies have reported that the host cells, such as human THP-1 macrophages, murine bone marrow-derived macrophages (BMDMs), and human microvascular endothelial cells (HMECs), secrete IFN-I during infection with *Rickettsia* sp. (6–9). Human THP-1 macrophages infected with a mildly pathogenic agent such as *Rickettsia parkeri*, *Rickettsia africae*, or *Rickettsia massiliae* produce different levels of interferon beta (IFN-β) to trigger different proteome signatures and differentially impact critical components of innate immune responses to interfere the replication of the SFG rickettsial agent (8). Also, *R. parkeri*-infected BMDMs produce IFN-β mediated by the DNA sensor cGAS, inducing the expression of interferon regulatory factor IRF5, which upregulates the expression of the genes encoding guanylate binding proteins and inducible nitric oxide synthase to inhibit the replication of *R. parkeri* (9). Moreover, under infection with *Rickettsia conorii,* a highly pathogenic agent, HMECs secrete IFN-β to activate signal transducer and activator of transcription protein 1 by phosphorylation in an autocrine/paracrine manner, promoting the expression of transcription factors interferon regulatory factor 7 (IRF7) and IRF9 and inhibiting the expression of suppressor of cytokine signaling protein SOCS1 and UBP43, which finally lead to the significant suppression of the intracellular replication of *R. conorii* (6, 7). However, the effect of IFN-I on the host cells infected with *R. rickettsii*, a highly pathogenic agent, has not been well known.

The host TRIM56 protein is reported to contribute to the IFN-I signal pathway and is a restriction factor of several RNA viruses (influenza virus, yellow fever virus, dengue virus, and bovine viral diarrhea virus) (10–12) and DNA viruses (Newcastle disease virus and herpes simplex virus) (13, 14) both in an E3 ligase-dependent and -independent manner. Furthermore, *Salmonella typhimurium* SopA HECT-type E3 ligase targets TRIM56 to stimulate RIG-I and MDA5 innate immune receptors, which subsequently modulates inflammatory responses (15). However, the role of TRIM56 in anti-bacterial infection remains vastly unexplored.

Here, we investigated the role of TRIM56 in the host innate immune response against *R. rickettsii* infection. Our results demonstrated that TRIM56 suppressed the intracellular replication of *R. rickettsii* in HeLa and THP-1 cells by inducing the production of IFN-β. This anti-microbial effect mediated by TRIM56 is dependent on the cysteine residues of the RING domain. Our findings not only gain insights into the mechanism of host innate immune response against *R. rickettsii* but also provide the potential therapeutic targets for RMSF.

## RESULTS

### *R. rickettsii* infection upregulates the expression of TRIM56 in host cells

**Fig 1:**
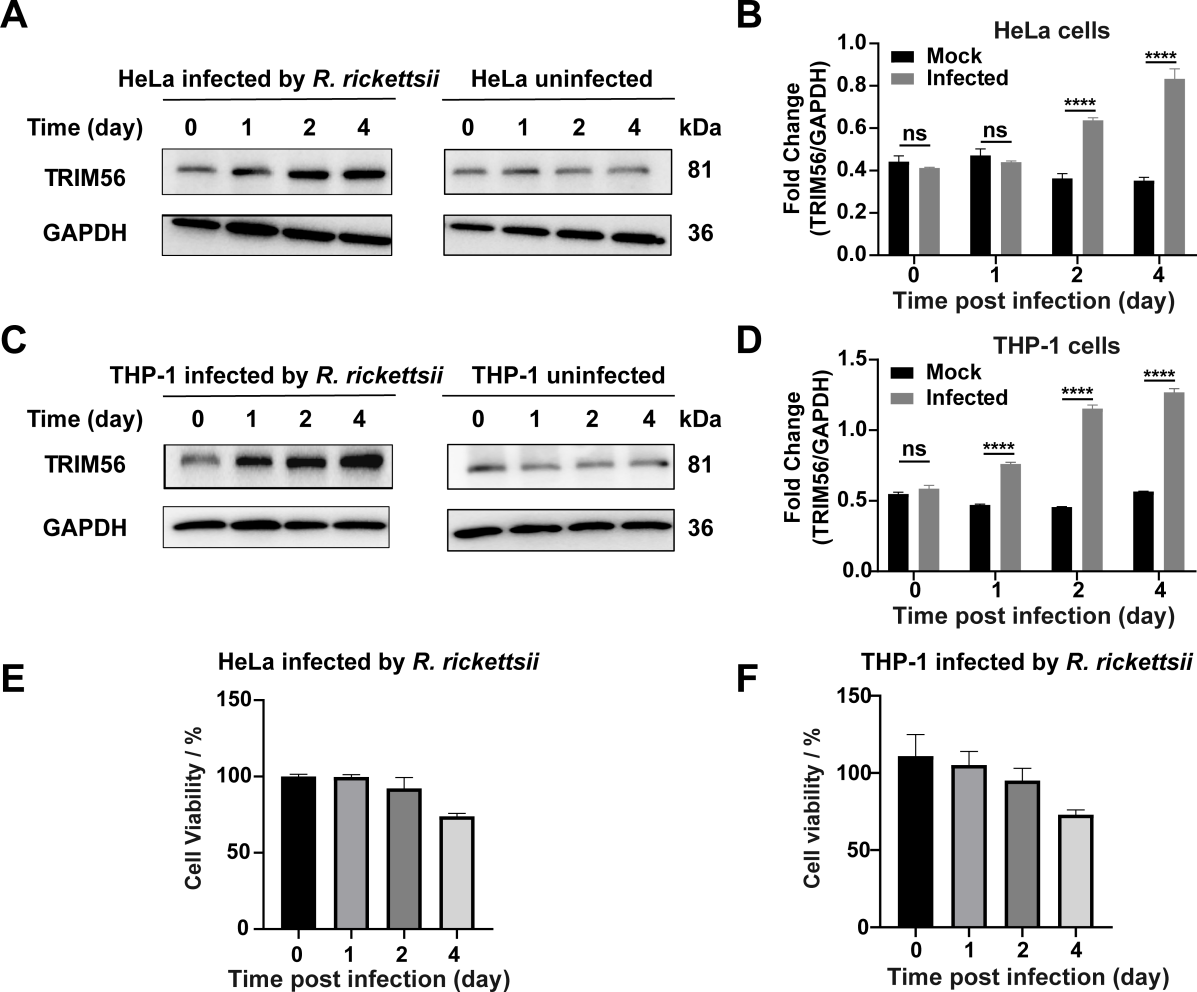
Upregulated expression of TRIM56 during *R. rickettsii* infection. HeLa cells were infected with *R. rickettsii* at an MOI of 1 or uninfected (**A**). Densitometry of HeLa cells was presented as fold change between the ratios of TRIM56/GAPDH (*n* = 3, mean ± SD) (**B**). PMA-differentiated THP-1 cells were infected with *R. rickettsii* at an multiplicity of infection (MOI) of 1 or uninfected (**C**). Densitometry of PMA-differentiated THP-1 cells was presented as a fold change between the ratios of TRIM56/GAPDH (*n* = 3, mean ± SD) (**D**). The cell viability of *R. rickettsii-*infected HeLa cells (**E**) and PMA-differentiated THP-1 cells (**F**) was measured by CCK-8. The infected cells were collected at 0, 1, 2, and 4 dpi to determine the protein levels of TRIM56 by Western blotting analysis, and GAPDH served as the loading control. The data presented were representative of at least three independent experiments. ^ns^*P* ≥ 0.05; ^****^P < 0.0001.

To explore the potential role of TRIM56, we first examined whether *R. rickettsii* infection alters TRIM56 expression in HeLa and THP-1 cells. HeLa and THP-1 were infected with *R. rickettsii*, respectively; the protein samples of HeLa and THP-1 cells were collected for analyses at 0, 1, 2, and 4 days post-infection (dpi). The TRIM56 mRNA level (Fig. S1) and protein levels were significantly increased following *R. rickettsii* infection in quantitative reverse transcription polymerase chain reaction (RT-qPCR) and Western blotting analysis in HeLa cells (Fig. 1A and B; Fig. S2A) and phorbol 12-myristate 13-acetate (PMA)-differentiated THP-1 cells (Fig. 1C and D; Fig. S2B) compared to uninfected control. Cell viability assessment revealed that around 30% of HeLa cells (Fig. 1E) and THP-1 cells (Fig. 1F) underwent cell death on the 4 dpi using the Cell Counting Kit-8 (CCK-8) assay, whereas cell viability remained above 90% during the initial 3 days. These results demonstrated that TRIM56 exhibited upregulation upon *R. rickettsii* infection in HeLa and THP-1 cells

### The alteration of TRIM56 expression level affects the intracellular replication of *R. rickettsii*

**Fig 2:**
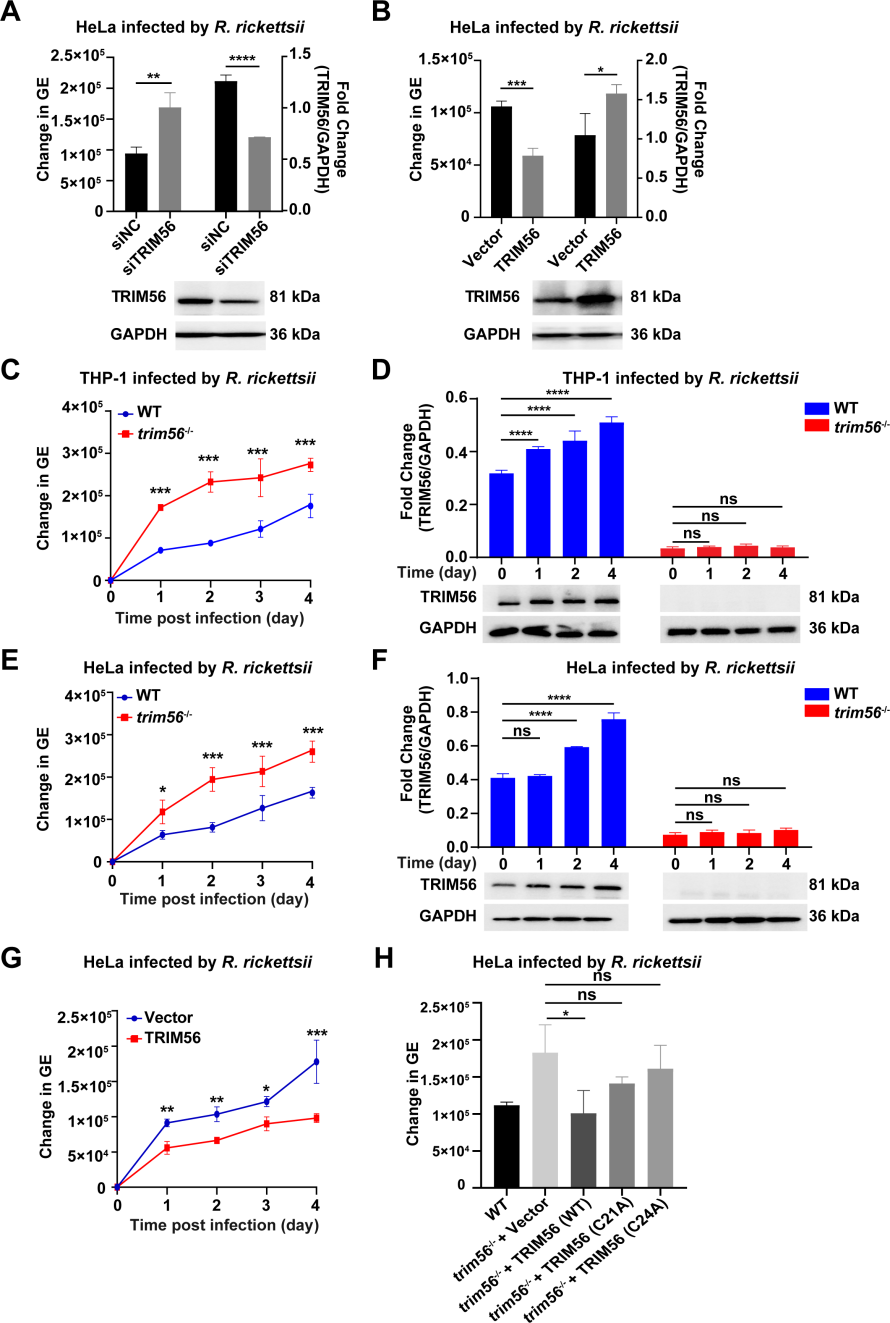
TRIM56 knockdown and overexpression, respectively, promote and inhibit *R. rickettsii* intracellular replication. (**A–B**) HeLa cells were transfected with the siTRIM56 (**A**) and pcTRIM56 (**B**), followed by *R. rickettsii* infection at an MOI of 1 for 4 days. The cells were washed and collected for DNA extraction to GE of *R. rickettsii* using qPCR, while protein lysates from the cells were subjected to Western blotting analysis to assess TRIM56 protein expression, and densitometry was presented as the fold change between the ratios of TRIM56/GAPDH (*n* = 3, mean ± SD). (**C–F**) *trim56*^−/−^ THP-1 cells (**C, D**) and *trim56*^−/−^ HeLa cells (**E, F**) were infected with *R. rickettsii* at an MOI of 1 for 0, 1, 2, 3, and 4 days. The cells were washed and collected for DNA extraction to quantify the GE of *R. rickettsii* using qPCR, while protein lysates from the cells were subjected to Western blotting analysis to assess TRIM56 protein expression, and densitometry was presented as the fold change between the ratios of TRIM56/GAPDH (*n* = 3, mean ± SD). (**G**) HeLa cells were transfected with vector and pcTRIM56, followed by *R. rickettsii* infection at an MOI of 1 for 0, 1, 2, 3, and 4 days. The cells were washed and collected for DNA extraction to quantify the GE of *R. rickettsii using* qPCR (*n* = 3, mean ± SD). (**H**) *trim56*^−/−^ HeLa cells were transfected with pcTRIM56 (WT), pcTRIM56 (C21A), pcTRIM56 (C24A), and vector, followed by *R. rickettsii* infection at an MOI of 1 for 4 days. The cells were washed and collected for DNA extraction to quantify the GE of *R. rickettsii* using qPCR (*n* = 3, mean ± SD). Data presented were representative of at least three independent experiments. ^ns^*P* ≥ 0.05; ^*^P < 0.05; ^**^P < 0.01; ^***^P < 0.001; ^****^P < 0.0001.

To investigate the impact of TRIM56 on *R. rickettsii* replication, HeLa cells were transfected with the small interfering RNA (siRNA) of TRIM56 (siTRIM56) and overexpression plasmid pcDNA3.1-hTRIM56-HA (pcTRIM56), while the nonsense siRNA (siNC) and plasmid vector pcDNA3.1-HA (vector) were set as controls. After *R. rickettsii* infection, total DNA was extracted, and the genomic equivalent (GE) of *R. rickettsii* DNA was quantitated using quantitative polymerase chain reaction (qPCR). The transfection of siTRIM56 led to a reduction in TRIM56 expression and an increased intracellular burden of *R. rickettsii* at 4 dpi (Fig. 2A; Fig. S3A). Conversely, the transfection of pcTRIM56 resulted in an enhanced TRIM56 expression and decreased bacterial loads at 4 dpi (Fig. 2B; Fig. S3B). To gain further insight into the anti-*R*. *rickettsii* role of TRIM56, the *trim56* gene was knocked out in HeLa and THP-1 cells. In order to understand the course of *R. rickettsii* infection regulated by TRIM56, wild-type (WT) cells, *trim56*^−/−^ cells, and TRIM56 overexpression cells were infected with *R. rickettsii*. The infected cells were collected at 0, 1, 2, 3, and 4 dpi for analyses of bacterial burden. Notably, *trim56*^−/−^ HeLa and THP-1 cells exhibited a complete lack of TRIM56 expression validated by Western blotting, accompanied by sustained high rickettsial load levels (Fig. 2C through F; Fig. S3C and D). TRIM56 overexpression cells displayed a sustained low level of rickettsial load (Fig. 2G), highlighting the positive role of TRIM56 in inhibiting intracellular replication of *R. rickettsii*. Furthermore, to elucidate the function of the RING domain characterized by E3 ligase activity of TRIM56, the TRIM56 point mutation plasmids containing cysteine residues at 21 and 24 to N-terminal of the RING domain (C21A and C24A) were transfected to *trim56*^−/−^ HeLa cells, but these mutations failed to suppress the replication of *R. rickettsii* in *trim56*^−/−^ HeLa cells at 4 dpi (Fig. 2H), suggesting that the inhibitory effect of TRIM56 on *R. rickettsii* replication was involved in 21 and 24 (C21 and C24) of the E3 catalytic site within the RING domain. Collectively, these findings provided evidence that TRIM56 exerted an inhibitory effect on *R. rickettsii* intracellular replication in a manner of C21 and C24 E3 catalytic site-dependent way.

### TRIM56 deficiency inhibits IFN-β transcription and secretion after *R. rickettsii* infection

**Fig 3:**
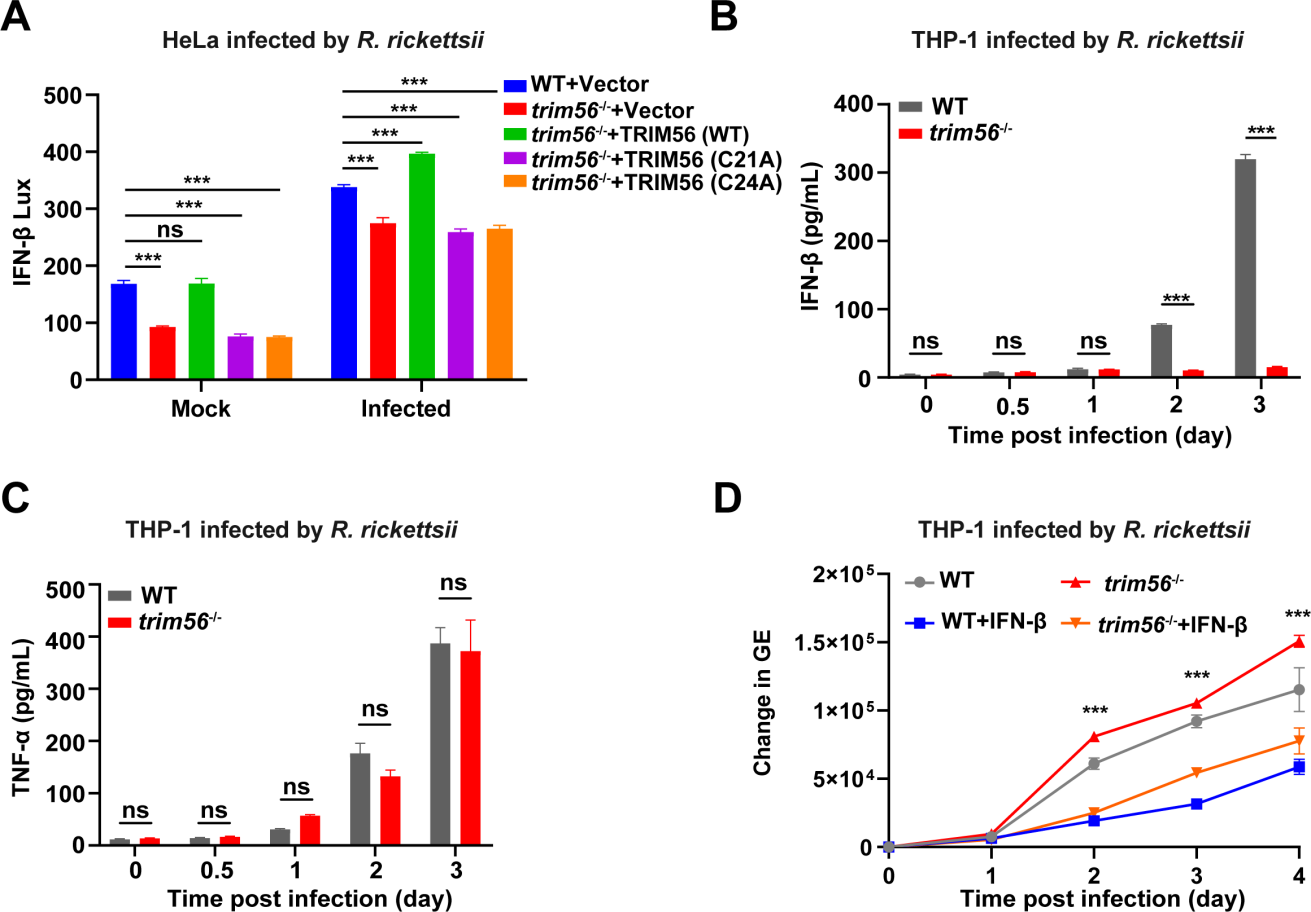
TRIM56 knockout inhibits IFN-β transcription and secretion during *R. rickettsii* infection. (**A**) The IFN-β levels (IFN-β Lux) of the different host cells. WT and *trim56*^−/−^ HeLa cells were co-transfected with the indicated plasmid pGL3.0-IFN β-luc plasmid, pcTRIM56 (WT), pcTRIM56 (C21A), pcTRIM56 (C24A), or vector, followed by infection with *R. rickettsii* at an MOI of 1 for 3 hours; then, the cells were lysed to detect the luminescence (*n* = 3, mean ± SD). (**B**) The concentration of IFN-β (pg/mL) from PMA-differentiated WT and *trim56*^−/−^ THP-1 cells infected by *R. rickettsii* at an MOI of 1 for 0, 0.5, 1, 2, and 3 days. (**C**) The concentration of TNF-α (pg/mL) from PMA-differentiated WT and *trim56*^−/−^ THP-1 cells infected by *R. rickettsii* at an MOI of 1 for 0, 0.5, 1, 2, and 3 days. (**D**) PMA-differentiated WT and *trim56*^−/−^ THP-1 cells were infected with *R. rickettsii* at an MOI of 1 for 3 hours, and then, the supernatant was discarded, and the cells were washed three times with phosphate-buffered saline (PBS), followed by treatment with 200 ng/mL IFN-β for 2 days. The cells were washed and collected for DNA extraction at 0, 1, 2, 3, and 4 dpi to quantify the GE of *R. rickettsii* using qPCR (*n* = 3, mean ± SD). Data presented were representative of at least three independent experiments. ^ns^*P* ≥ 0.05; ^*^P < 0.05; ^**^P < 0.01; ^***^P < 0.001. Statistical analyses in (B–D) were performed using two-way analysis of variance (ANOVA) with multiple comparisons, compared to WT.

Previous studies displayed that IFN-β caused a robust, dose-dependent restriction of *R. parkeri* growth in mouse bone marrow-derived macrophages (9). Studies also have shown that TRIM56 is involved in innate anti-viral immunity response, including the initiation of IFN response (16). To investigate the impact of TRIM56 on *R. rickettsii*-induced IFN-β response, WT and *trim56*^−/−^ HeLa cells, as well as THP-1 cells, were infected with *R. rickettsii*, and mRNA was collected to analyze the transcriptional level of IFN-β. As depicted in Fig. S4, the mRNA levels of IFN-β significantly increased following infection, with the WT cells exhibiting a significantly higher induction compared to *trim56*^−/−^ cells. To further analyze the role of TRIM56 in the regulation of the IFN-I pathway during *R. rickettsii* infection, WT and *trim56*^−/−^ HeLa cells were transfected with pGL3.0-IFN-β-luc plasmid with pcTRIM56, pcTRIM56 (C21A), pcTRIM56 (C24A), or vector, followed by *R. rickettsii* infection. As shown in Fig. 3A, the IFN-β promoter activity was decreased in *trim56*^−/−^ HeLa cells compared with the WT HeLa cells after transfection. In previous results, we found that the overexpression of TRIM56 inhibited the replication of *R. rickettsii* in HeLa cells. The transfection of the TRIM56 full-length plasmid group induced higher IFN-β expression levels, whereas the cells transfected with TRIM56 E3 ligase catalytic site mutation plasmids showed no significant changes in IFN-β expression. Therefore, we speculated that TRIM56 was able to affect the secretion of IFN-β after *R. rickettsii* infection. To verify whether TRIM56 deficiency affected IFN-β secretion, the supernatant of PMA-differentiated WT and *trim56*^−/−^ THP-1 cells infected by *R. rickettsii* was detected by the enzyme-linked immunosorbent assay (ELISA) at the 0, 0.5, 1, 2, and 3 dpi (Fig. 3B). As a result, WT THP-1 cells significantly increased IFN-β secretion, while *trim56*^−/−^ THP-1 cells did not during *R. rickettsii* infection. Meanwhile, TNF-α expression was not affected by *trim56* deletion (Fig. 3C). Next, to analyze the role of IFN-β during *R. rickettsii* infection, we treated the PMA-differentiated WT and *trim56*^−/−^ THP-1 cells with IFN-β (200 ng/mL) for 48 hours. Compared to the untreated groups, *R. rickettsii* replication was significantly decreased in IFN-β-treated WT and *trim56*^−/−^ THP-1 cells (Fig. 3D). Collectively, the above results suggested that TRIM56 deficiency specificity inhibited the transcription and secretion of IFN-β, thereby promoting the intracellular replication of *R. rickettsii*.

### TRIM56 positively regulates the cGAS-STING signaling pathway through ubiquitination of STING during *R. rickettsii* infection

**Fig 4:**
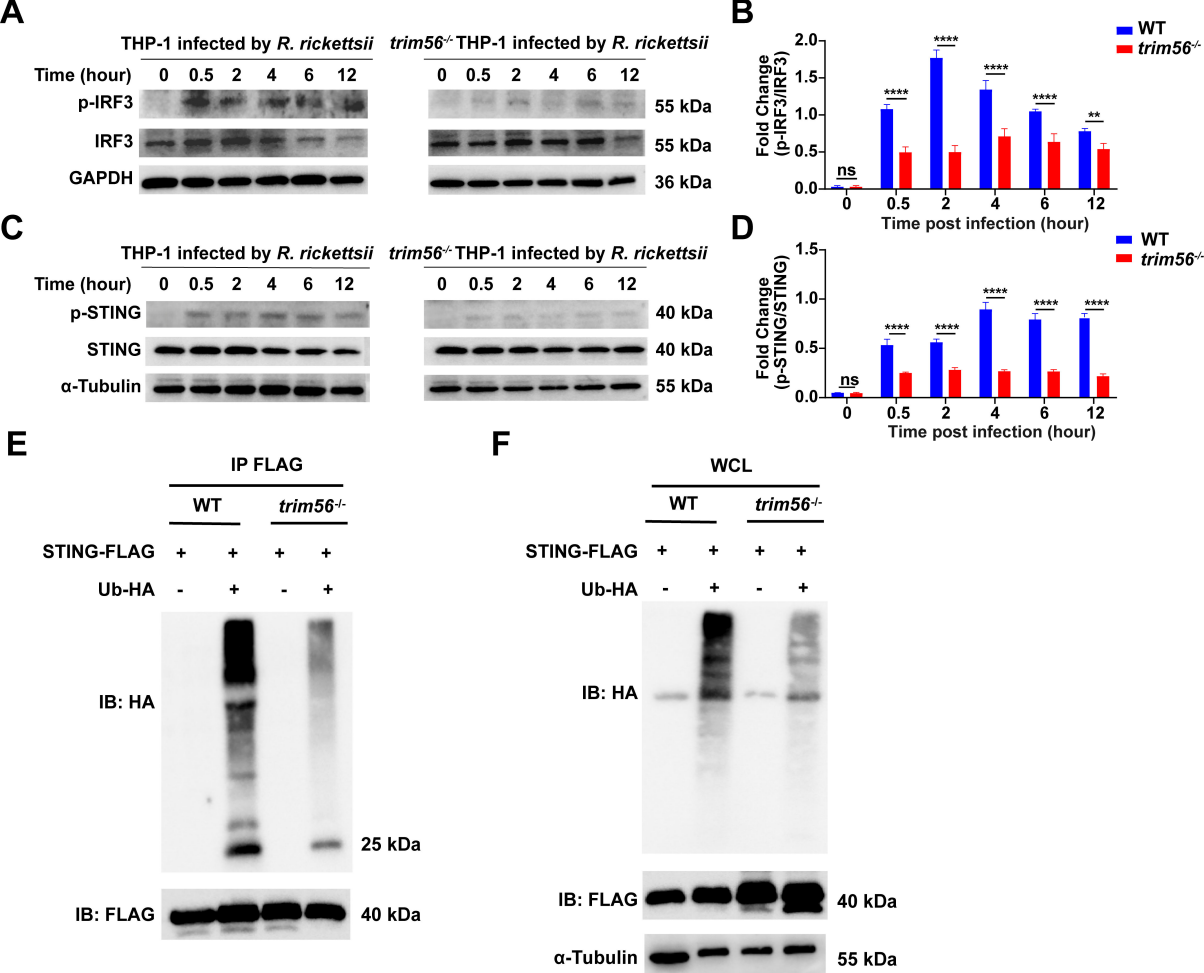
TRIM56 enhances the *R. rickettsii*-induced cGAS-STING signaling through ubiquitination of STING. (**A–B**) PMA-differentiated WT and *trim56*^−/−^ THP-1 cells were infected by *R. rickettsii,* and then, the infected cells were collected at 0, 0.5, 2, 4, 6, and 12 hours post-infection for Western blotting analysis, followed by probing with anti-p-IRF3, anti-IRF3, and anti-GAPDH, respectively. (**B**) Densitometry of (**A**) was presented as a fold change between the ratios of p-IRF3/IRF3 (*n* = 3, mean ± SD). (C–D) PMA-differentiated WT and *trim56*^–/−^ THP-1 cells were infected by *R. rickettsii,* and then, the infected cells were collected for Western blotting analysis, followed by probing with anti-p-STING, anti-STING, and GAPDH, respectively (**C**). Densitometry of (**C**) was presented as a fold change between the ratios of p-STING/STING (*n* = 3, mean ± SD) (**D**). **(E–F**) WT and *trim56*^–/–^ HeLa cells transfected with STING-FLAG and HA-Ub plasmids were infected with *R. rickettsii* at an MOI of 1 for 2 days, and then, the protein complexes were extracted from the cell lysates with anti-FLAG M2 beads. The immunoprecipitation with FLAG lysates (**E**) and whole-cell lysates (**F**) was probed with FLAG and HA antibodies. Data presented were representative of at least three independent experiments. ^ns^*P* ≥ 0.05; ^**^P < 0.01; ^****^P < 0.0001.

TRIM56 has been previously shown to directly modulate STING activity to induce type I IFN production in response to double-stranded DNA stimulation (13). To examine the activation of cGAS-STING signaling, PMA-differentiated WT and *trim56*^−/−^ THP-1 cells were infected with *R. rickettsii* for 0, 0.5, 2, 4, 6, and 12 hours, respectively, followed by Western blotting analysis. Compared with WT THP-1, the phosphorylation of IRF3 (p-IRF3) (Fig. 4A and B; Fig. S5A) and STING (p-STING) (Fig. 4C and D; Fig. S5B) in *trim56*^−/−^ THP-1 cells was significantly inhibited as evaluated by densitometry analysis. To further gain insights into how TRIM56 regulated the activation of STING during *R. rickettsii* infection, we investigated the potential of TRIM56-mediated ubiquitination of STING by co-immunoprecipitation (co-IP). The WT and *trim56*^−/−^ HeLa cells were transfected with STING-FLAG or Ub-HA, followed by *R. rickettsii* infection at an MOI of 1 for 2 days, and then, the cells were treated with MG132 and subjected to immunoprecipitation with an anti-FLAG antibody, followed by immunoblotting with an anti-HA antibody. We found that after knocking out TRIM56, the ubiquitination of STING was significantly reduced (Fig. 4E and F; Fig. S5C). These results demonstrated that TRIM56 E3 ligase positively regulated the cGAS-STING signaling pathway through the ubiquitination of the STING, finally leading to the influence production of IFN-β to suppress the intracellular replication of *R. rickettsii*.

## DISCUSSION

RMSF, a severe life-threatening tick-borne zoonotic disease with high fatality rates in people, is caused by *R. rickettsii*, the most pathogenic species of genus *Rickettsia*. If untreated, it can quickly progress into a life-threatening illness in people, with high fatality rates (17–19). Therefore, the search for effective anti-bacterial effectors is of great significance for the prevention and treatment of *R. rickettsii* infection. The activation of IFN-β signaling constitutes an important component of host defense mechanisms, yet the status of this pathway during *Rickettsia* infection of macrophage cells, the immune cells preferably targeted by pathogenic rickettsiae during human spotted fever syndromes, has so far remained an insufficient area of scientific inquiry (20, 21). Here, we report the TRIM56-mediated down-regulation of the intracellular replication of *R. rickettsii* with upregulation of IFN-β secretion by modulating cGAS-STING signaling pathways in HeLa and THP-1 cells, suggesting that TRIM56 also participated in the immune response against intracellular bacteria such as *R. rickettsii*.

This is in agreement with published evidence documenting the production of endogenous IFN-β by cultured human THP-1 macrophages, BMDMs, and HMECs in response to infection with mildly pathogenic typhus group species (6–9). Previous studies have also shown that *Rickettsia* species have undergone extensive genome reduction and are dependent on host processes for replication. IFN-β alters the cytosol to an uninhabitable environment for obligate microorganisms; this may occur through a combination of IFN-stimulated genes and alterations to metabolism (9). The present study unequivocally demonstrates that spotted fever group species *R. rickettsii* infection stimulates the expression and secretion of IFN-β in THP-1 cells compared to *trim56*^−/−^ cells, indicating that *R. rickettsii*-induced IFN-β expression likely occurs via TRIM56-mediated IFN-β secretion.

Upon infection, the presence of intracellular *Rickettsiae* is sensed by an as-yet unknown host surveillance system leading to the initiation of an IFN-β expression response; several intracellular signaling pathways are known to induce IFN-β expression during microbial infections (22–24). TLRs sense microbial DNA and RNA to activate transcription factors IRF3, resulting in the expression of IFN-β. Furthermore, RIG-1 and MDA5 in the cytosol detect double-stranded RNA from RNA viruses, triggering the induction of IFN-β. Additionally, the cGAS-STING signaling axis detects pathogenic DNA to trigger an innate immune reaction involving a strong IFN-I response against microbial infections. The previous study demonstrates that the typhus group species *R. parkeri*-induced IFN-I production of macrophages depends on cGAS, but the key host molecules mediating the signaling axis of the *Rickettsia*-induced cGAS-STING pathway remain to be elucidated (9). In the cGAS-STING signaling axis, IRF3 phosphorylation is essential for IFN-β production to initiate immune responses (25–28). Previous studies have shown that the induction of IFN-β in HUVECs infected with *Chlamydia pneumoniae* is reliant on IRF3 (29). In this study, the expression of the phosphorylation of IRF3 was significantly increased in THP-1 cells compared to *trim56*^−/−^ THP-1 cells upon *R. rickettsii* infection, suggesting that TRIM56 may facilitate the initiation of IRF3 phosphorylation in response to *R. rickettsii* infection.

The ubiquitination of STING, an important upstream regulator of IRF3 phosphorylation, is crucial on the cGAS-STING signaling axis (14, 30–32). According to reported studies, TRIM56 is capable of the ubiquitination of STING under the stimulation of virus infection (13, 33, 34). However, whether TRIM56 participates in the ubiquitination of STING under obligate intracellular bacterial infection is unclear. It has been reported that the facultative anaerobe *Salmonella typhimurium* effector SopA targets TRIM56 to inhibit its E3 ligase activity by occluding the E2-interacting surface, culminating in the suppression of RING ubiquitination to facilitate intracellular amplification of bacteria (15, 35). In this study, the ubiquitination of STING was reduced in *trim56*^−/−^ cells followed by *R. rickettsii* infection, demonstrating that the ubiquitination of STING in response to obligate intracellular bacteria *R. rickettsii* infection was in a TRIM56-dependent way.

Our study adds TRIM56 to the list of anti-pathogen TRIMs and provides novel insights for a better understanding of the anti-pathogen mechanisms of TRIM56, specifically against *Rickettsia*. Here, we also demonstrated that the depleting TRIM56 expression significantly reduced IFN-β mRNA expression during *R. rickettsii* infection, suggesting that TRIM56 might be a potential therapeutic target for infection with *R. rickettsii*. However, this study was limited to *in vitro* cell culture assays, and the *in vivo* role of TRIM56 remains unclear. In the future, animal-based experiments will be conducted to explore the *in vivo* role of TRIM56.

## MATERIALS AND METHODS

### Cell lines and bacteria

HeLa cells were purchased from ATCC and cultured in Dulbecco’s modified Eagle’s medium (DMEM) (Gibco, United States, catalog no. C11995500BT) supplemented with 10% fetal bovine serum (FBS) (Gibco, United States, catalog no. C10010500BT) and 1% penicillin–streptomycin (Gibco, United States, catalog no. 15140-122) at 37°C in a 5% CO_2_ incubator. Human monocytic leukemia cells (THP-1) were also obtained from ATCC and cultured in an RPMI 1640 medium supplemented with 10% FBS and 0.1% 2-mercaptoethanol (Gibco, United States, catalog no. 21985-023). HeLa-*trim56*^−/−^ (*trim56* knockout HeLa cell line) and THP-1-*trim56*^−/−^ (*trim56* knockout THP-1 cell line) were constructed by LiMan Biotechnology (Wuhan, China, catalog no. CL-0233) and cultured in DMEM and RPMI 1640 medium with 10% FBS and 0.1% 2-mercaptoethanol.

*R. rickettsii* was propagated in Vero cells for the preparation of stocks. *R. rickettsii* (Sheila Smith strain) was grown in Vero cells and isolated by isopycnic density gradient centrifugation in the BSL-3 laboratory as previously described (17, 36). Briefly, confluent monolayers of Vero cells grown in DMEM supplemented with 2% fetal bovine serum and 2 mM L-glutamine were infected with *R. rickettsii* at an MOI of 1 and then incubated in a 33°C incubator set at 5% CO_2_ until 50% of the monolayer was disrupted due to infection. The number of *R. rickettsii* organisms and viable rickettsial organisms in suspension was detected by qPCR (37) and plaque assay (38), respectively.

### *R. rickettsii* purification

Four to five days post-infection, the culture supernatant of Vero cells infected with *R. rickettsii* was discarded and replaced with PBS (Gibco, United States, catalog no. C10010500BT) in a culture bottle. The cells were then scraped using a cell scraper (Corning, United States, catalog no. 3010) and transferred into 50-mL centrifuge tubes, which were placed on ice. Subsequently, 8 mL of Vero cell suspension was aliquoted into 15-mL centrifuge tubes (Corning, United States, catalog no. 430790) and kept on ice. The UP-250 ultrasonic cell mill probe (Xinzhi, Ningbo, China) was used with ultrasonic intensity set at 30%, oscillating for 2 seconds followed by a 2-second pause, repeating this cycle for a duration of 1 minute (a total of 12 tubes of 15 mL). Low-speed centrifugation was performed at 4°C and 3,000 rpm for 10 minutes using a HITACHI himac CR 21GII centrifuge. The supernatant was collected from three centrifuge tubes in the low-speed centrifugation and then was added into one 50-mL centrifuge tube for the high-speed centrifugation at 12,000 rpm for 10 minutes at 4°C. The pellet was then resuspended with surose-phosphate-glutamate (SPG) and stored at −80°C.

### Plasmid and transfection

TRIM56 expression plasmid was constructed with routine molecular cloning techniques. The full-length human TRIM56 gene was synthesized by GeneScript (Nanjing, China) and cloned into pcDNA3.1-C-HA between the BamHI and XhoI restriction sites to create the mammalian expression construct pcDNA3.1-hTRIM56-HA. The point mutation plasmids of TRIM56 C21A and C24A were generated through homologous recombination. HeLa cells were seeded in a 12-well plate at a density of 1 × 10^5^ cells per well with 1 mL of complete growth medium; then, the recombinant plasmid was transiently transfected with Lipofectamine 3000 (Invitrogen, United States, catalog no. 100022052) according to the manufacturer’s instructions. The transfected cells were infected with *R. rickettsii* at an MOI of 1 for 3 hours in a 5% CO_2_ incubator at 33°C. Then, each cell well was washed three times with PBS and replaced with a fresh medium. Finally, the cells in wells were collected at different times post-infection, while whole DNA and protein samples were extracted from the collected cells for qPCR and Western blot analysis, respectively.

### RNA interference experiment

TRIM56 small interfering RNA (siTRIM56: 5′-GCAGCAGAAUAGUGUGGUATT-3′) and ControlSiRNA (siNC: 5′-UUCUUCGAACGUGUCACGUTT-3′) were synthesized by GenePharma (Suzhou, China). HeLa cells were inoculated in 12-well plates at 1 × 10^5^ cells per well in a 1 mL medium for 1 day. Then, the HeLa cells were transfected by siTRIM56 or siNC with RNAiMAX (Invitrogen, United States, catalog no. 100022052) at a final concentration of 20 nM according to the manufacturer’s instructions. The transfected cells were infected with *R. rickettsii* at an MOI of 1 for 3 hours in a 33°C 5% CO_2_ incubator; then, each cell well was washed three times with PBS and replaced with a fresh medium. The infected cells were collected at different times post-infection, while DNA and protein samples were extracted from the infected cells for qPCR and Western blotting analysis.

### Western blotting analysis

The cells were harvested and washed three times with PBS and then lysed in cell lysis buffer for Western and IP buffer (Beyotime, China, catalog no. P0013) with phenylmethanesulfonyl fluoride (PMSF, Beyotime, China, catalog no. ST506-2). The mixture was centrifuged at 12,000 *g* for 10 minutes, and the supernatant was collected. Samples were separated by SDS-PAGE and transferred to PVDF membranes (Millipore, United States, catalog no. IPVH00010). After blocking with 5% skim milk, the membranes were incubated with the indicated primary antibodies, followed by appropriate secondary antibodies. Blots were developed with an enhanced chemiluminescence kit (Gene protein link technology, China, catalog no. P06M31X). The primary antibodies used are as follows: rabbit polyclonal antibodies against TRIM56 purchased from Abcam (Cambridge, United Kingdom, catalog no. 10583), and rabbit polyclonal antibodies against IRF3 (catalog no. 4302S), p-IRF3 (catalog no. 4947S), STING (catalog no. 13647S), p-STING (catalog no. 50907S), and α-tubulin (catalog no. 2148S) were purchased from Cell Signaling Technology (United States). Mouse polyclonal antibodies against GAPDH were purchased from Proteintech (United States, catalog no. 60004-1-Ig); goat anti-rabbit (catalog no. P03S02S) and goat anti-mouse (catalog no. P03S01S) secondary antibodies were purchased from Gene protein link technology (China).

### RNA extraction and real-time PCR

Total RNA was isolated from cells by the PureLink RNA kit (Invitrogen, United States, catalog no. 1218302), and quantitative PCR reaction was monitored with the One-Step TB Green PrimeScript PLUS RT-PCR Kit (Takara, Japan, catalog no. RR096A). All the primers used in this study are listed in Table 1.

**TABLE 1:** Primers used in this study

Primer	Sequence 5′–3′
hTRIM56-F	GCCTGCATACCTACTGCCAAG
hTRIM56-R	GCAGCCCATTGACGAAGAAGT
hIFN-β-F	ATGACCAACAAGTGTCTCCTCC
hIFN-β-R	GGAATCCAAGCAAGTTGTAGCTC
hGAPDH-F	GGAGCGAGATCCCTCCAAAAT
hGAPDH-R	GGCTGTTGTCATACTTCTCATGG

### IFN-β luciferase reporter assay

The IFN-β luciferase reporter construct was purchased from Transvector (Chengdu, China). WT and *trim56*^–/–^ HeLa cells were seeded in a 12-well plate at a density of 1 × 10^5^ cells per well with 1 mL of complete growth medium. The next day, cells were transfected with IFN-β-Luc reporter plasmid, vector, pcTRIM56, pcTRIM56 (C21A), or pcTRIM56 (C24A) at a final concentration of 20 nM for 4 hours, followed by *R. rickettsii* infection at an MOI of 1 for 3 hours; then, the cells were lysed with the Dual-Luciferase Reporter Gene Assay Kit (Beyotime, China, catalog no. RG027) according to the manufacturer’s instructions, and luminescence was measured using the SpectraMax i3x microplate reader (Molecular Devices, United States).

### IFN-β treatment

WT and *trim56*^−/−^ THP-1 cells were seeded at a density of 1 × 10^5^ cells per well in 24-well plates in RPMI 1640 with 10% FBS supplemented with 162 nM PMA overnight. The next day, the media were exchanged for media without PMA supplemented. The cells were infected with *R. rickettsii* at an MOI of 1 for 3 hours at 33°C in a 5% CO_2_ incubator, then washed three times with PBS and replaced with a fresh medium containing 200 ng/mL IFN-β (MCE, catalog no. HY-P7024) for 48 hours. The cells were washed and collected for DNA extraction to quantify the GEs of *R. rickettsii* using qPCR.

### Enzyme-linked immunosorbent assay

Culture supernatants of uninfected and *Rickettsia*-infected THP-1 macrophages at the respective time points post-infection were collected, and IFN-β and TNF-α levels were measured with the human IFN-β ELISA Kit (Solarbio, China, catalog no. SEKH-0410) and human TNF-α ELISA Kit (Solarbio, China, catalog no. SEKH-0047), following the manufacturer’s instructions (8). Protein concentrations were determined based on the optical density at 450 nm using a BioTek Epoch2 microplate reader and compared to standard curves for purified IFN-β.

### Immunoprecipitation

Immunoprecipitation was performed following established protocols, as described previously (39). HeLa cells were seeded in a 12-well plate at a density of 1 × 10^5^ cells per well with 1 mL of complete growth medium, then co-transfected with pCMV-Flag-STING (Sino Biological, China, catalog no. HG29810-NF) and pCMV-HA-Ub (Ke Lei Biotechnology, China, catalog no. kl-zl-0513) for 12 hours followed by *R. rickettsii* infection at an MOI of 1 for 2 days, and 100 nM MG132 were added to cells for 8 hours before cells were lysed in IP buffer (Beyotime, China, catalog no. P0013). After pre-clearing with protein A/G agarose beads for 1 hour at 4°C, whole-cell lysates were used for immunoprecipitation with the indicated antibodies. A 50% slurry of FLAGM2 beads (Sigma, United States, catalog no. M8823) was added to 1 mL of cell lysates and incubated at 4°C for 6 hours. Immunoprecipitates were extensively washed five times with lysis buffer and eluted with SDS loading buffer by boiling for 5 minutes. The following immunoblot steps were consistent with Western blotting analysis.

### Statistical analysis

Statistical analyses and calculations were performed with GraphPad Prism 8 software. Student’s *t*-tests or two-way ANOVA was used to determine the difference, and *P*-values less than 0.05 were considered statistically significant.

## Data Availability

The raw data supporting the conclusions of this article will be made available by the authors, without undue reservation.

## SUPPLEMENTAL MATERIAL

Supplemental Figures S1-S5 spectrum.03695-23-s0001.docx Supplemental figures for RT-qPCR or immunoblots.

ASM does not own the copyrights to Supplemental Material that may be linked to, or accessed through, an article. The authors have granted ASM a non-exclusive, world-wide license to publish the Supplemental Material files. Please contact the corresponding author directly for reuse.

## References

[1] Parola P, Paddock CD, Socolovschi C, Labruna MB, Mediannikov O, Kernif T, Abdad MY, Stenos J, Bitam I, Fournier PE, Raoult D. 2013. Update on tick-borne rickettsioses around the world: a geographic approach. Clin Microbiol Rev 26:657–702. doi:10.1128/CMR.00032-13 24092850 PMC3811236

[2] Blanton LS. 2019. The rickettsioses: a practical update. Infect Dis Clin North Am 33:213–229. doi:10.1016/j.idc.2018.10.010 30712763 PMC6364315

[3] Jay R, Armstrong PA. 2020. Clinical characteristics of rocky mountain spotted fever in the United States: a literature review. J Vector Borne Dis 57:114–120. doi:10.4103/0972-9062.310863 34290155

[4] McNab F, Mayer-Barber K, Sher A, Wack A, O’Garra A. 2015. Type I interferons in infectious disease. Nat Rev Immunol 15:87–103. doi:10.1038/nri3787 25614319 PMC7162685

[5] Henry T, Kirimanjeswara GS, Ruby T, Jones JW, Peng K, Perret M, Ho L, Sauer JD, Iwakura Y, Metzger DW, Monack DM. 2010. Type I IFN signaling constrains IL-17A/F secretion by gammadelta T cells during bacterial infections. J Immunol 184:3755–3767. doi:10.4049/jimmunol.0902065 20176744 PMC2879132

[6] Colonne PM, Eremeeva ME, Sahni SK. 2011. Beta interferon-mediated activation of signal transducer and activator of transcription protein 1 interferes with Rickettsia conorii replication in human endothelial cells. Infect Immun 79:3733–3743. doi:10.1128/IAI.05008-11 21690236 PMC3165482

[7] Colonne PM, Sahni A, Sahni SK. 2013. Suppressor of cytokine signalling protein SOCS1 and UBP43 regulate the expression of type I interferon-stimulated genes in human microvascular endothelial cells infected with Rickettsia conorii. J Med Microbiol 62:968–979. doi:10.1099/jmm.0.054502-0 23558133 PMC3709555

[8] Curto P, Santa C, Cortes L, Manadas B, Simões I. 2021. Spotted fever group Rickettsia trigger species-specific alterations in macrophage proteome signatures with different impacts in host innate inflammatory responses. Microbiol Spectr 9:e0081421. doi:10.1128/spectrum.00814-21 34935429 PMC8693926

[9] Burke TP, Engström P, Chavez RA, Fonbuena JA, Vance RE, Welch MD. 2020. Inflammasome-mediated antagonism of type I interferon enhances Rickettsia pathogenesis. Nat Microbiol 5:688–696. doi:10.1038/s41564-020-0673-5 32123346 PMC7239376

[10] Liu B, Li NL, Shen Y, Bao X, Fabrizio T, Elbahesh H, Webby RJ, Li K. 2016. The C-terminal tail of TRIM56 dictates antiviral restriction of influenza A and B viruses by impeding viral RNA synthesis. J Virol 90:4369–4382. doi:10.1128/JVI.03172-15 26889027 PMC4836312

[11] Liu B, Li NL, Wang J, Shi PY, Wang T, Miller MA, Li K. 2014. Overlapping and distinct molecular determinants dictating the antiviral activities of TRIM56 against flaviviruses and coronavirus. J Virol 88:13821–13835. doi:10.1128/JVI.02505-14 25253338 PMC4248981

[12] Wang J, Liu B, Wang N, Lee YM, Liu C, Li K. 2011. TRIM56 is a virus- and interferon-inducible E3 ubiquitin ligase that restricts pestivirus infection. J Virol 85:3733–3745. doi:10.1128/JVI.02546-10 21289118 PMC3126137

[13] Tsuchida T, Zou J, Saitoh T, Kumar H, Abe T, Matsuura Y, Kawai T, Akira S. 2010. The ubiquitin ligase TRIM56 regulates innate immune responses to intracellular double-stranded DNA. Immunity 33:765–776. doi:10.1016/j.immuni.2010.10.013 21074459

[14] Seo GJ, Kim C, Shin WJ, Sklan EH, Eoh H, Jung JU. 2018. TRIM56-mediated monoubiquitination of cGAS for cytosolic DNA sensing. Nat Commun 9:613. doi:10.1038/s41467-018-02936-3 29426904 PMC5807518

[15] Kamanova J, Sun H, Lara-Tejero M, Galán JE. 2016. The Salmonella effector protein SopA modulates innate immune responses by targeting TRIM E3 ligase family members. PLoS Pathog 12:e1005552. doi:10.1371/journal.ppat.1005552 27058235 PMC4825927

[16] Shen Y, Li NL, Wang J, Liu B, Lester S, Li K. 2012. TRIM56 is an essential component of the TLR3 antiviral signaling pathway. J Biol Chem 287:36404–36413. doi:10.1074/jbc.M112.397075 22948160 PMC3476306

[17] Alhassan A, Liu H, McGill J, Cerezo A, Jakkula L, Nair ADS, Winkley E, Olson S, Marlow D, Sahni A, Narra HP, Sahni S, Henningson J, Ganta RR, Palmer GH. 2019. Rickettsia rickettsii whole-cell antigens offer protection against rocky mountain spotted fever in the canine host. Infect Immun 87:e00628-18. doi:10.1128/IAI.00628-18 30396898 PMC6346123

[18] Dai L, Meng J, Zhao X, Li Q, Shi B, Wu M, Zhang Q, Su G, Hu J, Shu X. 2022. High-spatial-resolution VOCs emission from the petrochemical industries and its differential regional effect on soil in typical economic zones of China. Sci Total Environ 827:154318. doi:10.1016/j.scitotenv.2022.154318 35257751

[19] Wilson CG, Alexander S, Hitch WJ, Lorenz-Miller L, Vaughan E, Fagan EB. 2022. Project ECHO in primary care: informing providers about COVID-19 and its impact on health care delivery. N C Med J 83:130–133. doi:10.18043/ncm.83.2.130 35256476

[20] Sahni A, Fang R, Sahni SK, Walker DH. 2019. Pathogenesis of rickettsial diseases: pathogenic and immune mechanisms of an endotheliotropic infection. Annu Rev Pathol 14:127–152. doi:10.1146/annurev-pathmechdis-012418-012800 30148688 PMC6505701

[21] Londoño AF, Scorpio DG, Dumler JS. 2023. Innate immunity in rickettsial infections. Front Cell Infect Microbiol 13:1187267. doi:10.3389/fcimb.2023.1187267 37228668 PMC10203653

[22] Kato H, Sato S, Yoneyama M, Yamamoto M, Uematsu S, Matsui K, Tsujimura T, Takeda K, Fujita T, Takeuchi O, Akira S. 2005. Cell type-specific involvement of RIG-I in antiviral response. Immunity 23:19–28. doi:10.1016/j.immuni.2005.04.010 16039576

[23] Perry AK, Chen G, Zheng D, Tang H, Cheng G. 2005. The host type I interferon response to viral and bacterial infections. Cell Res 15:407–422. doi:10.1038/sj.cr.7290309 15987599

[24] Chang H, Hou P, Wang X, Xiang A, Wu H, Qi W, Yang R, Wang X, Li X, He W, Zhao G, Sun W, Wang T, He DC, Wang H, Gao Y, He H. 2023. CD97 negatively regulates the innate immune response against RNA viruses by promoting RNF125-mediated RIG-I degradation. Cell Mol Immunol 20:1457–1471. doi:10.1038/s41423-023-01103-z 37978243 PMC10687259

[25] Stierschneider A, Wiesner C. 2023. Shedding light on the molecular and regulatory mechanisms of TLR4 signaling in endothelial cells under physiological and inflamed conditions. Front Immunol 14:1264889. doi:10.3389/fimmu.2023.1264889 38077393 PMC10704247

[26] Honda K, Yanai H, Negishi H, Asagiri M, Sato M, Mizutani T, Shimada N, Ohba Y, Takaoka A, Yoshida N, Taniguchi T. 2005. IRF-7 is the master regulator of type-I interferon-dependent immune responses. Nature 434:772–777. doi:10.1038/nature03464 15800576

[27] Savitsky D, Tamura T, Yanai H, Taniguchi T. 2010. Regulation of immunity and oncogenesis by the IRF transcription factor family. Cancer Immunol Immunother 59:489–510. doi:10.1007/s00262-009-0804-6 20049431 PMC11030943

[28] Majumdar T, Chattopadhyay S, Ozhegov E, Dhar J, Goswami R, Sen GC, Barik S. 2015. Induction of interferon-stimulated genes by IRF3 promotes replication of Toxoplasma gondii. PLoS Pathog 11:e1004779. doi:10.1371/journal.ppat.1004779 25811886 PMC4374777

[29] Buss C, Opitz B, Hocke AC, Lippmann J, van Laak V, Hippenstiel S, Krüll M, Suttorp N, Eitel J. 2010. Essential role of mitochondrial antiviral signaling, IFN regulatory factor (IRF)3, and IRF7 in Chlamydophila pneumoniae-mediated IFN-β response and control of bacterial replication in human endothelial cells. J Immunol 184:3072–3078. doi:10.4049/jimmunol.0902947 20154210

[30] Liu S, Cai X, Wu J, Cong Q, Chen X, Li T, Du F, Ren J, Wu YT, Grishin NV, Chen ZJ. 2015. Phosphorylation of innate immune adaptor proteins MAVS, STING, and TRIF induces IRF3 activation. Science 347:aaa2630. doi:10.1126/science.aaa2630 25636800

[31] Wang F, Alain T, Szretter KJ, Stephenson K, Pol JG, Atherton MJ, Hoang H-D, Fonseca BD, Zakaria C, Chen L, et al.. 2016. S6K-STING interaction regulates cytosolic DNA-mediated activation of the transcription factor IRF3. Nat Immunol 17:514–522. doi:10.1038/ni.3433 27043414 PMC4917298

[32] Ni G, Konno H, Barber GN. 2017. Ubiquitination of STING at lysine 224 controls IRF3 activation. Sci Immunol 2:eaah7119. doi:10.1126/sciimmunol.aah7119 28763789 PMC5656267

[33] Ozato K, Shin DM, Chang TH, Morse HC. 2008. TRIM family proteins and their emerging roles in innate immunity. Nat Rev Immunol 8:849–860. doi:10.1038/nri2413 18836477 PMC3433745

[34] Fu L, Zhou X, Jiao Q, Chen X. 2023. The functions of TRIM56 in antiviral innate immunity and tumorigenesis. Int J Mol Sci 24:5046. doi:10.3390/ijms24055046 36902478 PMC10003129

[35] Fiskin E, Bhogaraju S, Herhaus L, Kalayil S, Hahn M, Dikic I. 2017. Structural basis for the recognition and degradation of host TRIM proteins by Salmonella effector SopA. Nat Commun 8:14004. doi:10.1038/ncomms14004 28084320 PMC5241803

[36] Gong W, Wang P, Xiong X, Jiao J, Yang X, Wen B. 2015. Enhanced protection against Rickettsia rickettsii infection in C3H/HeN mice by immunization with a combination of a recombinant adhesin rAdr2 and a protein fragment rOmpB-4 derived from outer membrane protein B. Vaccine 33:985–992. doi:10.1016/j.vaccine.2015.01.017 25597943

[37] Duan C, Meng Y, Wang X, Xiong X, Wen B. 2011. Exploratory study on pathogenesis of far-eastern spotted fever. Am J Trop Med Hyg 85:504–509. doi:10.4269/ajtmh.2011.10-0660 21896812 PMC3163874

[38] Weinberg EH, Stakebake JR, Gerone PJ. 1969. Plaque assay for Rickettsia rickettsii. J Bacteriol 98:398–402. doi:10.1128/jb.98.2.398-402.1969 4977475 PMC284828

[39] Fu M, Liu Y, Wang G, Wang P, Zhang J, Chen C, Zhao M, Zhang S, Jiao J, Ouyang X, Yu Y, Wen B, He C, Wang J, Zhou D, Xiong X, Luo Z. 2022. A protein-protein interaction map reveals that the Coxiella burnetii effector CirB inhibits host proteasome activity. PLoS Pathog 18:e1010660. doi:10.1371/journal.ppat.1010660 35816513 PMC9273094

