# Supplementary material for: TRIM56-mediated production of type I interferon inhibits intracellular replication of Rickettsia rickettsii
Source: Microbiol Spectr. 2024 Feb 15;12(4):e03695-23. doi: 10.1128/spectrum.03695-23 (PMC10986528; doi:10.1128/spectrum.03695-23)
Supplement: Supplemental Figures S1-S5 — Supplemental figures for RT-qPCR or immunoblots. [file spectrum.03695-23-s0001.docx]

Supplementary Material


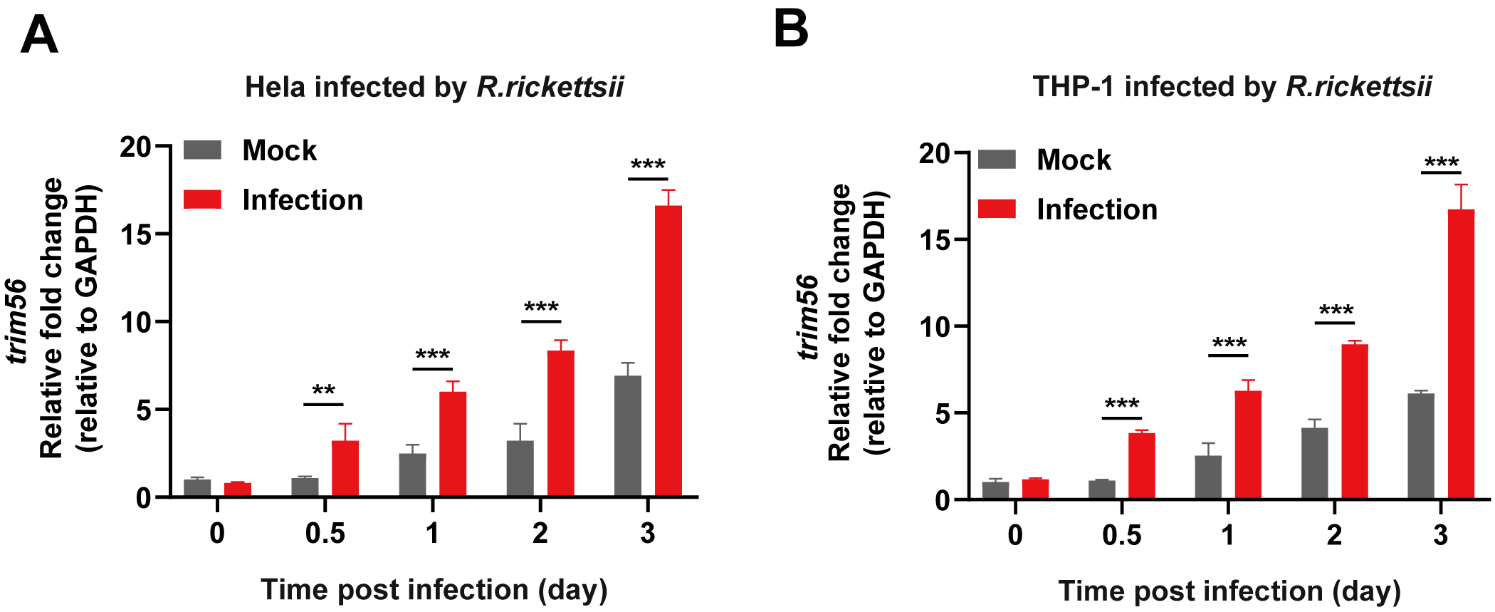


**Supplementary Figure 1.** Up-regulated mRNA level of TRIM56 during *R. rickettsii* infection. Hela (A) and THP-1 (B) cells were infected with *R. rickettsii* at an MOI of 1 for 0, 0.5, 1, 2, and 3 days, and cells were collected for RNA extraction to determine the TRIM56 mRNA level by RT-qPCR. Results were normalized to GAPDH and fold changes were calculated by comparing the normalized changes at different days post-infection to day.


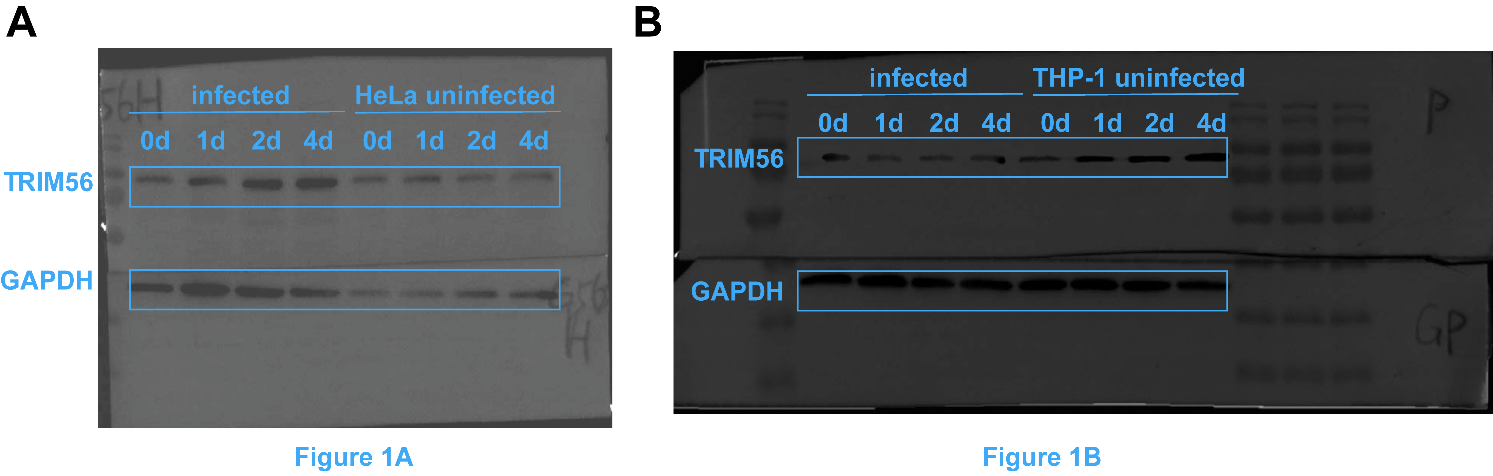


**Supplementary Figure 2.** Full immunoblots of Figure 1.


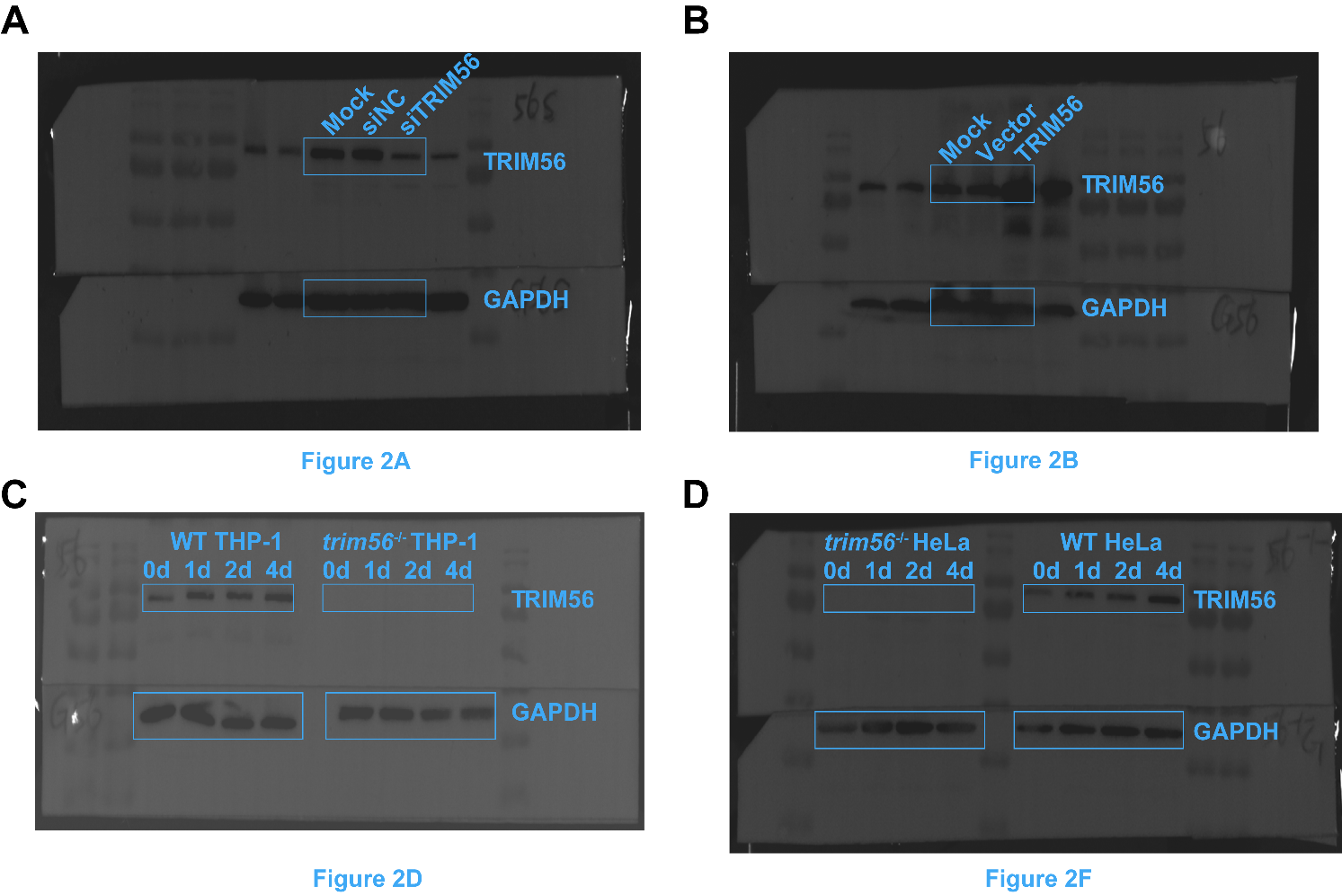


**Supplementary Figure 3.** Full immunoblots of Figure 2.


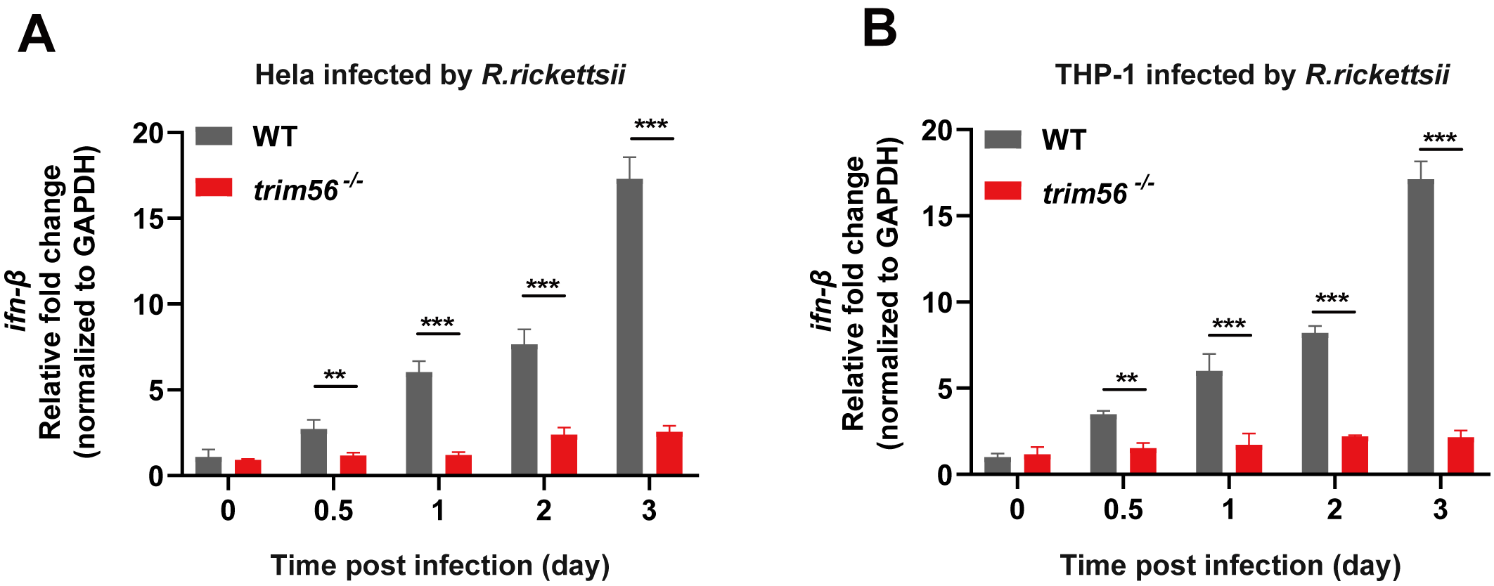


**Supplementary Figure 4.** Up-regulated mRNA level of IFN-β during *R. rickettsii* infection. WT THP-1 (A) and *trim56*^-/-^ THP-1 (B) cells were infected with *R. rickettsii* at an MOI of 1 for 0, 0.5, 1, 2, and 3 days, and cells were collected for RNA extraction to determine the IFN-β mRNA level by RT-qPCR. Results were normalized to GAPDH and fold changes were calculated by comparing the normalized changes at different days post-infection to day.


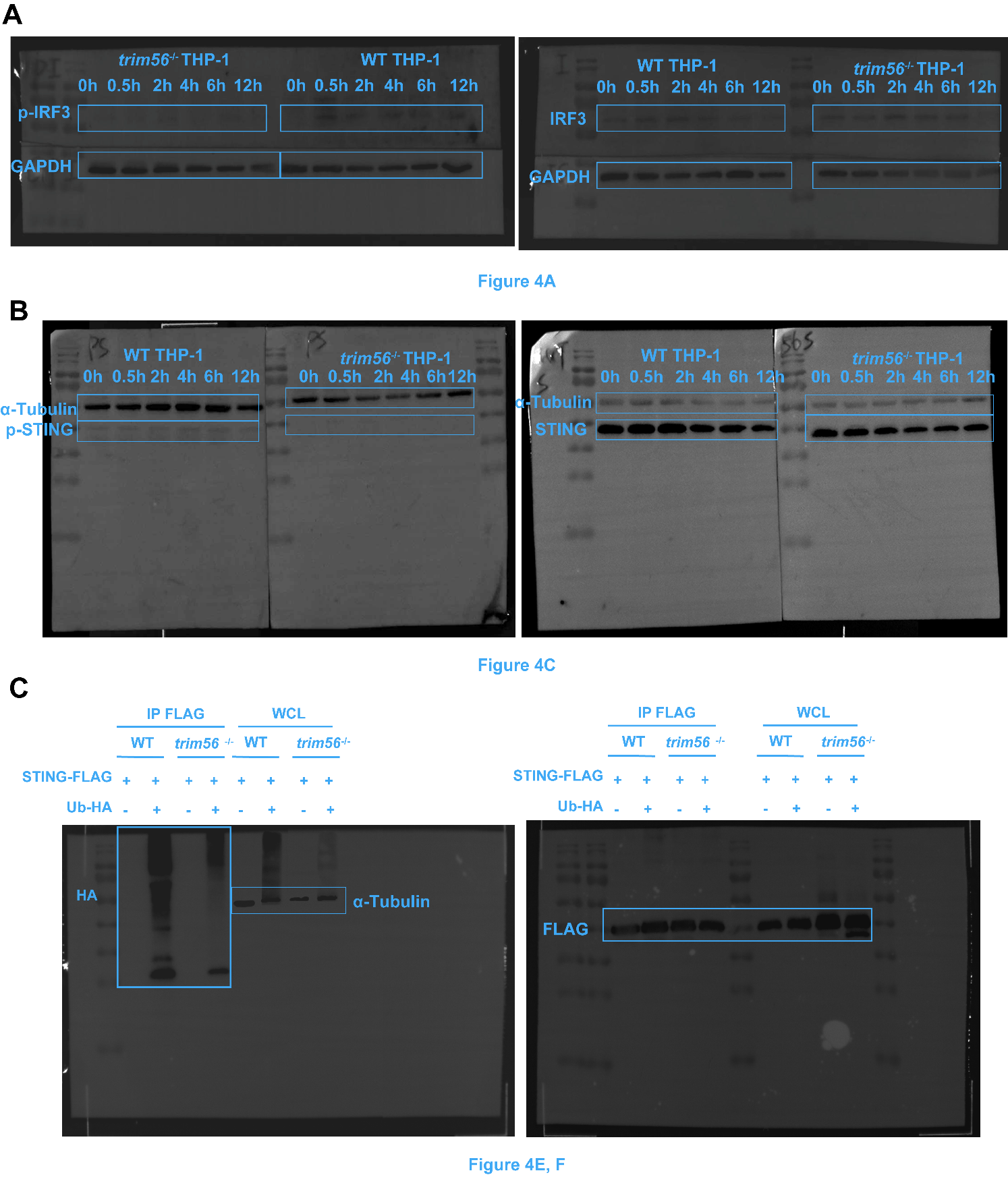


**Supplementary Figure 5.** Full immunoblots of Figure 4.
